# Genomic Mutations in SARS-CoV-2 Genome following Infection in Syrian Golden Hamster and Associated Lung Pathologies

**DOI:** 10.3390/pathogens12111328

**Published:** 2023-11-08

**Authors:** Gudepalya Renukaiah Rudramurthy, Chakenahalli N. Naveenkumar, Kumaraswamy Bharathkumar, Radha K. Shandil, Shridhar Narayanan

**Affiliations:** Foundation for Neglected Disease Research (FNDR), Plot No. 20A, KIADB Industrial Area, Bengaluru 561203, Karnataka, India; naveenkumar.cn@fndr.in (C.N.N.); bharathkumar.k@fndr.in (K.B.); rk.shandil@fndr.in (R.K.S.); shridhar.narayanan@fndr.in (S.N.)

**Keywords:** ancestral Wuhan strain, antiviral, COVID-19, mutation, NGS, S protein, SARS-CoV-2, SNP

## Abstract

The continuous evolution of the SARS-CoV-2 virus led to constant developments and efforts in understanding the significance and impacts of SARS-CoV-2 variants on human health. Our study aimed to determine the accumulation of genetic mutations and associated lung pathologies in male and female hamsters infected with the ancestral Wuhan strain of SARS-CoV-2. The present study showed no significant difference in the viral load between male and female hamsters and peak infection was found to be on day four post infection in both sexes of the animals. Live virus particles were detected up to 5 days post infection (dpi) through the TCID-50 assay, while qRT-PCR could detect viral RNA up to 14 dpi from all the infected animals. Further, the determination of the neutralizing antibody titer showed the onset of the humoral immune response as early as 4 dpi in both sexes against SARS-CoV-2, and a significant cross-protection against the delta variant of SARS-CoV-2 was observed. Histopathology showed edema, inflammation, inflammatory cell infiltration, necrosis, and degeneration of alveolar and bronchial epithelium cells from 3 dpi to 14 dpi in both sexes. Furthermore, next-generation sequencing (NGS) showed up to 10 single-nucleotide polymorphisms (SNPs) in the SARS-CoV-2 (ancestral Wuhan strain) genome isolated from both male and female hamsters. The mutation observed at the 23014 position (Glu484Asp) in the SARS-CoV-2 genome isolated from both sexes of the hamsters plays a significant role in the antiviral efficacy of small molecules, vaccines, and the Mabs-targeting S protein. The present study shows that either of the genders can be used in the pre-clinical efficacy of antiviral agents against SARS-CoV-2 in hamsters. However, considering the major mutation in the S protein, the understanding of the genetic mutation in SARS-CoV-2 after passing through hamsters is crucial in deciding the efficacy of the antiviral agents targeting the S protein. **Importance:** Our study findings indicate the accumulation of genomic mutations in SARS-CoV-2 after passing through the Syrian golden hamsters. Understanding the genomic mutations showed that either of the hamster genders can be used in the pre-clinical efficacy of antiviral agents and vaccines.

## 1. Introduction

Coronaviruses (CoVs) belonging to the family Coronaviridae and the order Nidovirales are genetically diverse and enveloped viruses, consisting of four genera, alpha, beta, gamma, and delta. Coronaviruses are capable of infecting multiple animal species and several cross-species transmission incidences have been identified [1]. Humans are known to be infected by only alpha and beta CoVs, and the majority of these infections are thought to be due to zoonotic transmission [2,3,4,5]. The ongoing pandemic caused by severe acute respiratory syndrome coronavirus 2 (SARS-CoV-2), known as coronavirus disease 2019 (COVID-19), was reported in early December 2019, in Wuhan, China [6,7]. The SARS-CoV-2 belonging to beta coronaviruses is more contagious [8] and continuous mutations in the spike protein led to the emergence of multiple variants such as B.1.1.7 (alpha), B.1.351 (beta), B.1.1.28.1/P.1 (gamma), B.1.617.2 (delta), B.1.617.1 (or kappa), and B.1.1.529 (omicron), which are circulating globally in the ongoing COVID-19 pandemic [9,10], and many different lethal variants will keep emerging in the future. The World Health Organization designated the B.1.617.2 (delta) variant as a variant of concern (VOC) and mutations in the spike protein have increased the binding efficiency to the ACE2 receptor, the transmissibility, secondary attack rates, and the escape from immunity and neutralization [11]. 

The clinical symptoms and severity of SARS-CoV-2 infection are highly variable with a significant mortality, and populations with underlying medical conditions are highly vulnerable [6]. Millions of human lives have been claimed since the emergence of COVID-19 with a catastrophic impact, and this has pushed the economy of the world into a significantly bad shape. Several different vaccines have been developed at a rapid pace and have been given approval for emergency use by the respective government agencies. The swift vaccination programs and management strategies across the globe helped in managing the severity of SARS-CoV-2 in subsequent infection waves. However, the development of specific antivirals is another area of focus and urgent need, and further, the specific antivirals or therapies against any human CoVs are not yet available [12,13]. 

A key strategy to minimize the effect of the COVID-19 pandemic on human health and to protect from the ongoing pandemic is the development of effective antivirals and vaccines. Preclinical studies involving animals play a critical role in the determination of efficacy of the biologics and antivirals and help further take up the clinical studies based on the preclinical data. Hence, developing and validating suitable animal infection models play a crucial role in the preclinical study of antiviral inhibitors and vaccine efficacy. However, the absence of appropriate receptors for many human viruses in animals impedes the development of suitable animal infection models. Small mammals such as Syrian hamsters (*Mesocricetus auratus*) are known to express ACE-2 receptors and interact with the receptor binding domain (RBD) of SARS-CoV-2 [14] and mediate entry [15]. Further, hamsters are susceptible to several respiratory virus infections such as SARS-CoV, influenza, and SARS-CoV-2, and have been suitably used as infection models for antiviral and vaccine efficacy testing [16,17,18]. The pathogenicity, severity of lung disease, and tissue tropism during SARS-CoV-2 infection in hamsters have been evaluated and hamsters serve as suitable mammalian infection models for COVID-19 [19]. 

In the present study, we evaluated the SARS-CoV-2 replicative ability, course of infection, and lung pathology in male and female Syrian golden hamsters with an ancestral Wuhan Strain of SARS-CoV-2. Further, the mutation capability of SARS-CoV-2 after passing through male and female hamsters was evaluated to determine the accumulation of genomic mutations in SARS-CoV-2 post-animal infection. The viral load in the lungs was determined through a standard plaque assay and quantitative real-time PCR. This study included the identification of differences in histopathological changes in the lungs of both males and females infected with the Wuhan strain of SARS-CoV-2. Furthermore, the RNA isolated from the virus stock (SARS-CoV-2) used in animal infection and the viral RNAs extracted from the infected animal tissues were sequenced to determine any mutations in the SARS-CoV-2 genome. The SARS-CoV-2 infection study was carried out in a high-containment (BSL-3) facility. 

## 2. Materials and Methods

### 2.1. Cell Lines and Virus Propagation

The Vero-E6 cell (C1008) (African green monkey kidney epithelial cell) was obtained from Elabscience Biotechnology Inc. (Cat no. EP-CL-0491) and maintained in Dulbecco’s modified Eagle’s medium (DMEM) supplemented with 10% fetal bovine serum (FBS), an antibiotic antimycotic solution containing penicillin (100 units/ml), streptomycin (100 µg/ml), and amphotericin B (0.25 µg/ml). 

SARS-CoV-2 isolates, USA-WA1/2020 (ancestral Wuhan) (Cat No; NR-52281) and B.1.617.2 (delta) (Cat No; NR- NR-55611), used in the present study were obtained from BEI Resources. The virus stocks were prepared by propagating the virus strains in Vero E6 cells by following the standard protocol [20,21]. The virus stocks were titrated using a gold-standard plaque assay [20] to determine the plaque forming units (PFU/ml) and 50% tissue culture infective dose (TCID50/ml) and stored at −80 °C in small aliquots [22]. The TCID50/ml of the stocks was determined using the Reed and Muench method [23]. Furthermore, the viral RNA was extracted from both stocks and subjected to next-generation sequencing (NGS) to confirm the genome sequence of SARS-CoV-2. 

### 2.2. SARS-CoV-2 Infection in Syrian Golden Hamsters

The male and female hamsters were used in the present study to understand the replicative capability, pathology, and mutation rates of SARS-CoV-2 (ancestral Wuhan strain). The Syrian golden hamsters (males and females) used in the present study were obtained from ACTREC (Advanced Centre for Treatment, Research and Education in Cancer, Mumbai). The health status of the animals and body weights were regularly monitored during the quarantine period for 7 days before being used in this study. The studies were conducted in our BSL-3 containment facility. The males and females were separately grouped (n = 5) and acclimatized at the BSL-3 facility for 7 days before the study initiation. The animals were anesthetized with ketamine at 150 mg/kg body weight and Xylazine at 10 mg/Kg body weight through the intraperitoneal route and intranasally infected (100 µL) with SARS-CoV-2 virus, USA-WA1/2020 (ancestral Wuhan) at 10^5^ TCID50 per animal. The uninfected group (Mock control) in each study was intranasally given 100 µL of DMEM to mimic the infection group.

The animals were sacrificed on different days post-infection (dpi) of 2, 3, 4, 5, 7, and 14 dpi to understand the pathogenesis and immune response during SARS-CoV-2 infection. The body weight of the animals was recorded before the sacrifice at each time point. The whole lungs were collected from the animals and the right lobe of the lungs was used to determine the viral load, while the left lobes were fixed in 10% buffered formalin for histopathology study. The mock control groups were terminated at the last time point (14 dpi). The animals were euthanized and sacrificed by following the standard approved protocol and the institutional CPCSEA number is FNDR-FB-83-PD-2021.

### 2.3. Determination of Viral Load from the Lung Samples

The infectious virus load in the lung tissues of the control and infected groups was determined using standard TCID-50 assay and real-time quantitative PCR. The tissue samples were added with sterile PBS at a 1:2 ratio (*w*/*v*) and homogenized using a Tissue Homogenizer (BIOSPEC PRODUCTS INC., USA Cat No; 985370EUR-07) for 10 seconds and then the homogenate was clarified through centrifugation. The viral load from the supernatant was determined through standard TCID-50 assay and is expressed as TCID-50/lung tissue. 

Further, the viral RNA was extracted from all the tissue homogenates using QIAamp Viral RNA Mini Kit (Qiagen) by following the manufacturer’s instructions. Quantitative real-time PCR (qRT-PCR) was performed to determine the genomic viral RNA using US-2019-nCoV Assays for the detection of SARS-CoV-2 RNA (Sigma Darmstadt, Germany, Cat no.: CDA00011). The viral RNA per lung tissue was calculated and compared to determine the course of infection in male and female hamsters with two different variants of SARS-CoV-2. 

### 2.4. Next-Generation Sequencing (NGS) to Study Mutation in the Viral RNA

The NGS study was conducted to determine any mutations in the viral RNA after passing through hamsters in comparison to virus stocks used in animal inoculation. For this study, the viral RNA was extracted from the virus stock used in the infection of hamsters and from the lung homogenates of infected male (n = 1) and female (n = 1) groups at 4 dpi (Table 1). The extracted samples were confirmed for the presence of SARS-CoV-2 viral RNA using qRT-PCR. The whole-genome sequencing of the isolated RNA samples was conducted using the NextSeq500 platform (https://www.illumina.com/systems/sequencing-platforms/nextseq.html, accessed on 5 November 2023). The consensus sequence was extracted from each sample, and a single-nucleotide polymorphism (SNP) was identified using the mpileup utility of Samtools (version; v 0.1.18). The most likely lineages to the sample consensus sequences were assigned using the Pangolin tool. 

### 2.5. Immune Response against SARS-CoV-2 Infection in Hamsters

The neutralizing antibodies in the serum samples of infected hamsters were determined through the Microneutralization Test (MNT) to understand the immune response elicited by hamsters against SARS-CoV-2 infection. The blood samples were collected at time points of 2, 3, 4, 5, 7, and 14 dpi from all the animals from different groups, and serum was separated, heat-inactivated at 56 °C for 1 h, and preserved at −80 °C until further use. The neutralizing antibody titer in the sera sample was determined using MNT against SARS-CoV-2, USA-WA1/2020 (ancestral Wuhan) to determine the immunological protection. Further, the neutralizing antibodies against SARS-CoV-2, delta variant (B.1.617.2), were estimated to determine the cross-protection elicited by SARS-CoV-2 (ancestral Wuhan) infection in hamsters. In brief, the serum samples were serially diluted and incubated with the respective SARS-CoV-2 variants at 100 TCID-50 for 1 hour at 37 °C. Upon completion of the incubation, the virus load was determined to calculate the 50% virus neutralization. To summarize, the ancestral strain (Wuhan strain)-infected animal sera samples were tested against the SARS-CoV-2 (ancestral Wuhan and delta variant) to understand the earliest protection and cross-protection elicited during SARS-CoV-2 infection in animals.

### 2.6. Lung Histopathology

The lungs were collected from infected groups and mock control group animals on the day of sacrifice at different time points. The images of whole lungs were captured to observe any gross pathological changes in comparison to the control group. The left lobes of the lungs were perfused with 10% buffered formalin and subjected to histopathological analysis. In brief, the samples were processed for paraffin embedding after fixing in 10% buffered formalin. Subsequently, thin sections (3 µm) of paraffin blocks were prepared and mounted on a glass slide. The sections were then stained with hematoxylin and eosin and the images were captured at 10× and 60× magnification. 

### 2.7. Statistical Analysis

The data collected from this study such as body weight and viral load were analyzed using Graph pad Prism software (Version; 9.3.1, Boston, MA, USA). The data were statistically analyzed using a two-tailed unpaired *t*-test to determine the significance between the genders and variants of SARS-CoV-2 infection.

## 3. Results

### 3.1. Change in Body Weight Remained Similar in Both Sexes

**Figure 1:**
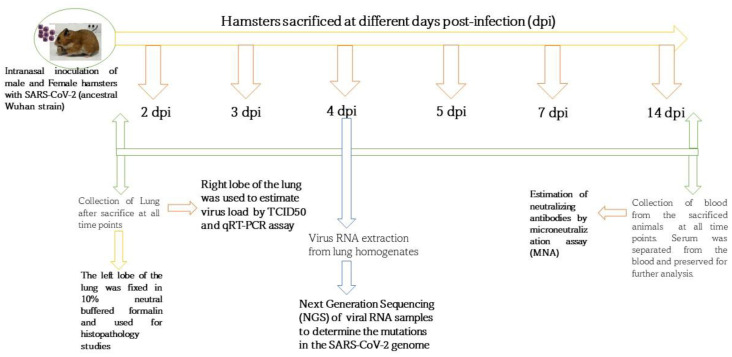
Schematic representation depicting the study design; the Syrian golden hamsters (male and female) were infected with SARS-CoV-2 (ancestral Wuhan). The animals were sacrificed on different days post-infection (dpi) to collect the lung and blood samples for viral load estimation (lung) and neutralizing antibody estimation (serum).

**Figure 2:**
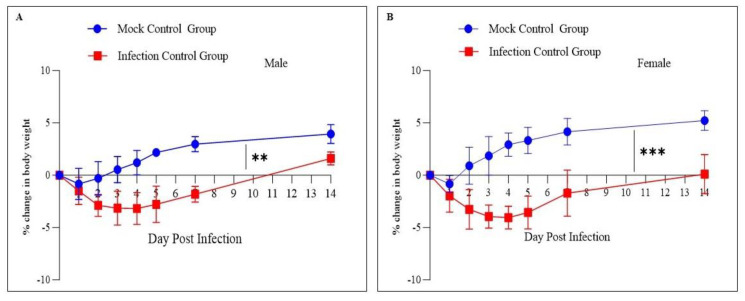
Percentage body weight change. (**A**,**B**): male and female hamsters infected with the SARS-CoV-2 (ancestral Wuhan strain), respectively. The body weight of the animals was recorded from day 0 (before infection) until day 14 post-infection. The infected animals showed up to a 5% decrease in body weight up to 5 dpi; however, mock animals showed an increase in body weight. Later, the animals gained body weight with a significant difference in body weight between mock and infected groups in both sexes of the animals. *** *p* < 0.001 and ** *p* < 0.01 using a two-tailed unpaired *t*-test.

The body weight of the study animals was measured before the sacrifice at every time point to determine the change in body weight. The schematic diagram depicting the study design and time points is shown in Figure 1. The mock control group animals showed a decrease in body weight on day one post-infection followed by recovery in the body weight on subsequent days. The infected group showed a reduced body weight (up to 5%) until 5 dpi and subsequently recovered the body weight. The change in body weight showed a significant difference between the mock and infected groups and a similar trend was observed in both sexes of the animals (Figure 2A,B). 

### 3.2. Whole-Genome Sequencing of Viral RNA Showed Mutations in the Spike Protein

The viral RNA isolated from the virus stock (used in the infection of hamsters) and from the infected lung samples of male (n = 1) and female (n = 1) hamsters were subjected to whole-genome sequencing using next-generation sequencing (NGS), to identify mutations in the SARS-CoV-2 genome. The whole genome of the stock virus (ancestral Wuhan) used in the infection of hamsters was sequenced and compared with the reference sequence SARS-CoV- 2, isolate Wuhan-Hu-1, complete genome (NC_045512.2). The comparison showed 8 SNPs, 5 of which were in the spike (S) protein gene, 2 of which were in the ORF1ab, and 1 of which was in the ORF8 (Table 1). The comparison details of SNPs and respective changes in the amino acid sequences are given in Table 2. The viral RNA genomes isolated from the lungs of hamsters (male and female) were compared with the stock virus (used in infection) to identify the SNPs. The comparison showed 10 SNPs in both male and female hamsters (Table 1). The mutation sites remain similar between the viral genomes isolated from male and female hamsters; however, at position 22296, viral RNA from the male hamster showed a mutation from G to A with a protein change from Arg245His, while the viral RNA from the female hamster did not show any mutation (Table 3). 

### 3.3. Lung Viral Load Revealed No Significant Difference between Male and Female Hamsters

**Figure 3:**
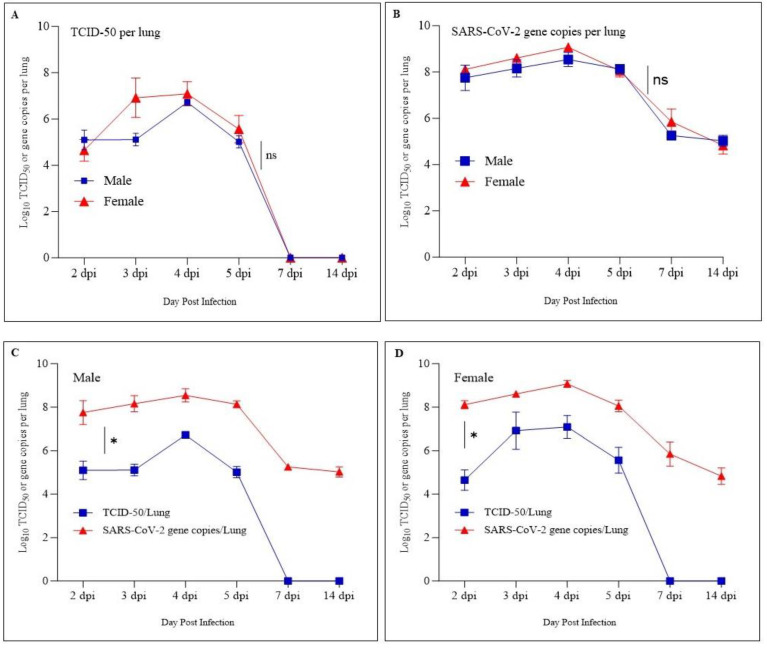
**Determination of infectious viral particles and viral RNA from lung homogenates:** male and female hamsters infected with SARS-CoV-2 (ancestral Wuhan strain). Both TCID-50 and qRTPCR assay showed peak viral load at 4 dpi regardless of the sex of the animal with no significant difference (**A**,**B**). Infectious virus particles were not detected using TCID50 assay at 7 dpi and onward, while qRTPCR detected viral RNA up to 14 dpi with a significant difference between the assays (**C**,**D**). * *p* < 0.1, and ns, non-significant using a two-tailed unpaired *t*-test.

The infectious SARS-CoV-2 virus particles and SARS-CoV-2 RNA copies in the lung samples of infected animals were determined using the TCID-50 assay and qRT-PCR, respectively. The TCID-50 assay showed a peak average viral load of log_10_ 6.0 and qRT-PCR showed average viral RNA copies of log_10_ 8.0 at 4 dpi in both male and female hamsters. The viral load/RNA copies’ data showed no significant difference among male and female hamsters (Figure 3A,B). The TCID-50 assay could not detect SRAS-CoV-2 virus particles at 7 dpi and 14 dpi from both males and females. However, the qRT-PCR assay could detect SARS-CoV-2 RNA both at 7 dpi and 14 dpi with an approximate viral RNA copy of log_10_ 5.0 (Figure 3C,D). 

### 3.4. Immune Response against SARS-CoV-2 Has Been Detected in Infected Hamsters as Early as 4 dpi

**Figure 4:**
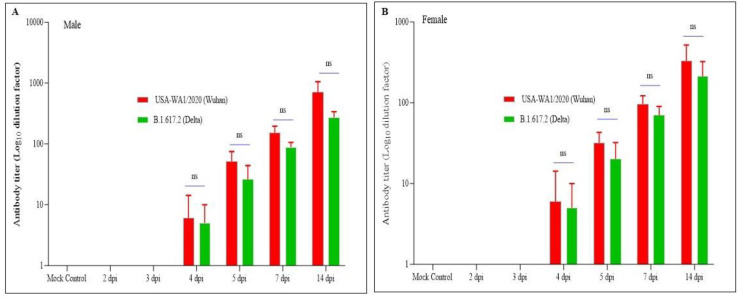
**Determination of neutralizing antibodies from infectious hamster serum samples:** (**A**,**B**): male and female hamsters infected with SARS-CoV-2 (ancestral Wuhan strain), respectively. Neutralizing antibodies were detected as early as 4 dpi regardless of the sex of the animal with no significant difference. Further, the cross-protecting antibodies were observed regardless of the sex of the animals with no significant difference. ns, non-significant using a two-tailed unpaired *t*-test.

The antibody titers in the serum samples of infected animals at different time points were estimated against SARS-CoV-2 variants to determine the early time point at which the immune response was elicited in the hamsters. The neutralizing antibodies in serum samples were determined using the microneutralization assay. The neutralizing antibodies against SARS-CoV-2 were observed at 4 dpi against both variants in both males and females. The hamsters (male and female) infected with the SARS-CoV-2 (ancestral Wuhan strain) showed a similar neutralizing antibody titer against the delta variant from 4 dpi and onward. Further, no significant difference was observed between male and female hamsters in the antibody neutralization capability against both the ancestral Wuhan strain and the delta variant (Figure 4A,B).

### 3.5. Significant Lung Pathology Has Been Observed as Early as 2 dpi in SARS-CoV-2-Infected Hamsters

**Figure 5:**
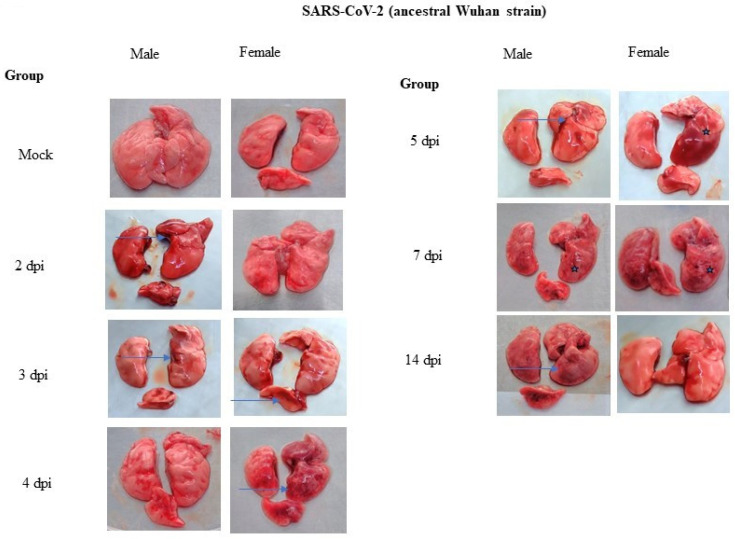
Gross pathology of the lungs. Gross pathological changes were observed at 3 dpi and onward in all the groups. The gross pathology was found to be severe at 4 dpi and onward to 7 dpi and reduced at 14 dpi in all the groups. 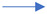 Represent the areas of congestion and hemorrhage and 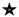 represent lung consolidation.

The SARS-CoV-2-induced progression of respiratory disease was detected through the evaluation of lung histopathological changes. The gross pathological examination of the lungs showed no abnormal gross lesions until 2 dpi in both male and female hamsters infected with the SARS-CoV-2 (ancestral Wuhan strain). At 3 dpi, slight generalized edema with areas of focal congestion was observed in all the groups. The lung pathology was found to be severe including marked inflammation, multifocal areas of hemorrhage, congestion, and edema at 4 dpi and onward to 7 dpi in all the groups. However, a reduced lung pathology was observed at 14 dpi in all the groups (Figure 5). 

**Figure 6:**
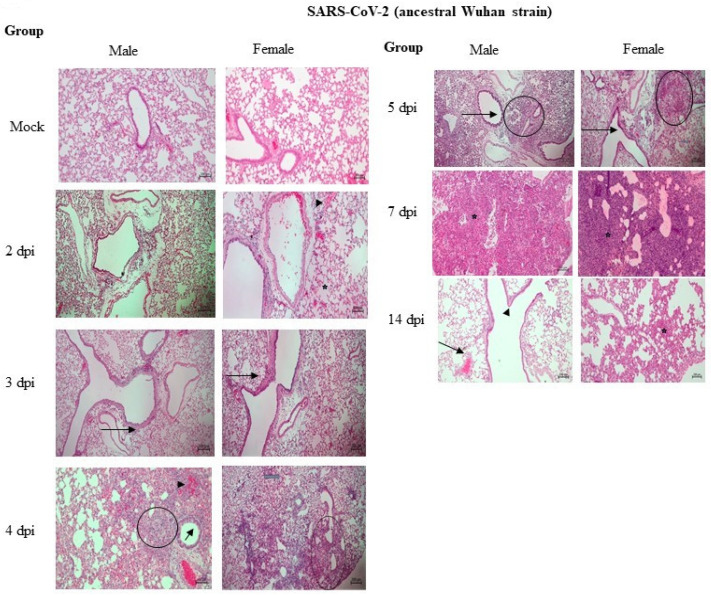
Histopathology of the lungs. Thin sections of the tissue samples were stained by hematoxylin and eosin and then the images were captured at 10× and 60× magnification, respectively, using an eyepiece and objective. The histopathological features such as inflammation and necrosis started as early as 2 dpi, showing an acute inflammatory response with diffuse alveolar damage, hemorrhages, congestion (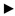), and an accumulation of eosinophilic edematous exudate (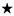). Moderate to marked broncho-interstitial pneumonia with alveolar damage and inflammatory cell infiltration (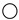), hemorrhage (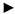), and hyperplasia of the bronchial epithelium (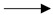) at 3 and 4 dpi in both male and female hamsters. Severe broncho-interstitial pneumonia with a marked increase in lung cellularity with hyperplasia of Type II alveolar cells (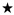) was observed at 7 dpi. Mild congestion (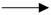), infiltration of inflammatory cells (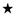), and mild hyperplasia of the bronchial epithelium (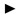) were observed at 14 dpi.

The histopathological examination revealed minimal to mild inflammation with focal inflammatory cell infiltration, mild necrosis, degeneration of alveolar and bronchial epithelium cells, and peribronchial and perivascular inflammatory cell infiltration with vascular congestion at 2 dpi and 3 dpi in both male and female hamsters (Figure 6). 

At 4 dpi and 5 dpi, the lung samples showed multifocal areas of inflammation, moderate to marked broncho-interstitial pneumonia, and an accumulation of mononuclear inflammatory cells like neutrophils, lymphocytes, and macrophages. Furthermore, moderate alveolar and bronchial epithelium degeneration, necrosis with cell debris, and inflammatory cells in the alveolar and bronchial spaces were prominent. Vascular changes like edema, mild endotheliosis, congestion, and areas of hemorrhage were observed in alveoli, and in peribronchial and perivascular areas, mild to moderate bronchial and alveolar epithelial hyperplasia with hyperplasia of type II alveolar epithelia were observed (Figure 6).

The lung pathology at 7 dpi in both males and females showed a similar lung pathology such as marked to severe multifocal broncho-interstitial pneumonia with a marked increase in lung cellularity. Further, the inflammatory cell infiltrates and exudate identified at 3 to 5 dpi were largely replaced by proliferative bronchiolar and alveolar epithelia. Moreover, marked alveolar and bronchial epithelium degeneration and marked hyperplasia of Type II alveolar cells were observed (Figure 6). At 14 dpi, the lungs showed a minimal focal infiltration of inflammatory cells and congestion. However, hyperplasia of the bronchial epithelium cells was observed in all the animals at 14 dpi (Figure 6). 

## 4. Discussion

Syrian hamsters (*Mesocricetus auratus*) have been used as animal infection models for many respiratory and non-respiratory viruses such as influenza virus, SARS-CoV, and Japanese encephalitis virus [24]. Golden Syrian hamsters that are susceptible to almost every strain of SARS-CoV-2 are being explored in the study of SARS-CoV-2-related pathogenesis, vaccine, and antiviral efficacy testing [11,24,25]. The present study is designed to determine the viral replication and pathogenesis associated with SARS-CoV-2 infection in male and female hamsters. Further, this study was aimed at the determination of the accumulation of genetic mutations in the ancestral Wuhan strain of SARS-CoV-2 in male and female hamsters. 

The body weight measurement at different time points showed a body weight loss of up to 5% in both sexes of the animals up to 4 dpi followed by a gain in body weight. Previous studies have shown that the SARS-CoV-2 infection in hamsters is associated with clinical manifestations such as reduced body weight up to 7 dpi followed by recovery [19,26,27]. Further, it has been observed that the change in body weight in hamsters is associated with virus inoculum dose rather than the age of the animal [28,29]. The estimation of SARS-CoV-2 viral RNA through qRT-PCR and viral particles through the TCID-50 assay showed peak infection at 4 dpi in both male and female hamsters, and the present results agree with the previous studies [19,26]. None of the groups showed live virus particles at 7 dpi and 14 dpi through the TCID-50 assay; however, qRT-PCR could detect the viral RNA at 7 dpi and 14 dpi, and this is due to the presence of viral RNA in the lungs even after the virus clearance [24]. The TCID-50 assay detects live virus particles, whereas qRT-PCR detects viral RNA from both live and dead virus particles.

The immune response against SARS-CoV-2 infection has been evaluated through the determination of the neutralizing antibody titer from the serum samples collected at different time points. The neutralizing antibody titers were detected as early as 4 dpi in both males and females from infected hamsters’ sera (Figure 4). The neutralizing antibody titer remains slightly higher against homologous infected sera compared to cross-reactive neutralizing antibodies. Further, the difference in antibody titer was not observed between the sexes of the animals. In the earlier study, no significant difference in antibody titers was observed amongst variants from B.1.1.7-infected serum samples at 14 and 28 dpi [30]. The present study showed the early onset of humoral immune response and the cross-protection elicited against different variants of SARS-CoV-2. 

The gross lung pathology showed inflammation, multiple hemorrhages, and edema as early as 3 dpi up to 14 dpi in all the animals except mock control animals with severe gross lung pathology. Previous studies have shown that the SARS-CoV-2-infection-related lung pathologies in hamsters resemble those of human lung pathologies including inflammation, alveolar epithelial injury, and pneumonitis [30,31,32]. The histopathology study showed inflammation as early as 2 dpi in both male and female hamsters. The onset of inflammatory lesions as early as 2 dpi indicates acute lung damage and increased severity by 4 dpi up to 7 dpi (Figure 6). Previous studies have shown widespread necrosis and inflammation at 2 dpi and 4 dpi [33,34]; further, alveolar and interstitial macrophage infiltration and type II hyperplasia were observed at 7 dpi [35]. The evaluation of lung histopathology revealed no significant differences among the sexes of the animals. A study by Plunkard et al. showed largely consistent lung pathologies across different variants of SARS-CoV-2 [27]. 

The NGS of the SARS-CoV-2 genome was conducted to determine the SNPs between the virus stock used in infection versus the virus passed through both male and female hamsters. The NGS data showed 8 SNPs in the SARS-CoV-2 (ancestral Wuhan strain) virus stock propagated in the Vero E6 cells and used in the infection of hamsters. Out of 8 SNPs, 5 were in the spike protein gene, 2 were in the ORF1ab, and 1 was in ORF8 from female and male hamsters (Table 2). Earlier studies showed the accumulation of adaptive mutations in the spike protein of the SARS-CoV-2 genome after different passages in Vero E6 cells [36,37]. The NGS data showed 10 SNPs in the SRAS-CoV-2 (ancestral Wuhan strain) genome isolated from both female and male hamsters compared to the virus stock used in the infection of hamsters. The number of mutations in the spike protein was found to be higher compared to other proteins such as ORF1ab. Mutations in the S protein of SARS-CoV-2 at key residues play a significant role in the interaction with the ACE2 receptor. The E484 of the Receptor binding domain (RBD) has been identified as an important position, and a change in the amino acid from E to K, Q, or P is known to reduce the antibody neutralization and change receptor affinity [38,39,40,41]. Further, the exposure of SARS-CoV-2 to mAbs has identified E484K as an escape mutation and only this mutation reduced the neutralizing ability of the combination of monoclonal antibodies (Mabs) [42,43]. Several earlier studies demonstrated a partial or complete loss of antiviral activity due to mutations in the SARS-CoV-2 genome [44]. An earlier study by Sonnleitner et al. reported the immune escape mutations in the SARS-CoV-2 genome from immunocompromised patients using NGS and the results from this study suggest that specific mutations may serve as targets for the development of vaccines and treatments for COVID-19 in the future [45]. Furthermore, a majority (five SNPs) of the mutations that were found in the virus stock (propagated in Vero E6 cells and used in the infection of hamsters) were replaced by the wildtype variant (NC_045512.2) in the viral genome isolated from hamsters in both sexes (Table S1). A significant difference in mutation and associated protein changes was not observed in the SARS-CoV-2 genome isolated from male and female hamsters, except for a mutation at position 22296 (Arg245His) in males, while in females, no mutation was observed (Table 1 and Table 2). The lack of significant difference in the genetic mutations in the SARS-CoV-2 (ancestral Wuhan) genome in male and female hamsters along with similar lung pathologies provides the basis for using either of the sexes in pre-clinical efficacy testing involving small molecules, vaccines, and MAbs. However, the identification of the mutation sites in the S protein after passing through hamsters is crucial in the preclinical efficacy study of antiviral agents targeting the S protein including MAbs and small-molecule inhibitors in hamsters. 

Major features such as sequence generation, assembly, alignment, variant identification, annotation, prioritization, and visualization are all shared by NGS [46,47,48] and require stringent quality control to warrant the validity of results. Earlier studies have shown variations in the frequency of SNPs ranging from 2.72% to 96.74% and variable mapping rates in the viral genome and this depends on the quality of the sample/storage conditions [49,50]. Further, the low-frequency variants reflect contaminations between samples, intra-host heterogeneity, coinfection with different viral strains, or PCR polymerase errors [51,52]. Although the present study provides information on the mutation sites in SARS-CoV-2 after passing through the hamsters, understanding the genome mutations in the virus used in the preclinical efficacy study is crucial in determining the antiviral effect of vaccines, small molecules, and Mabs including antiviral drug resistance. 

## 5. Conclusions

The continued emergence of new variants of SARS-CoV-2 emphasizes the concerted efforts and need to understand the SARS-CoV-2 pathology. Our study findings indicate the accumulation of genomic mutations in SARS-CoV-2 after passing through the Syrian golden hamsters with no differences among the sexes of the hamsters. Hence, understanding the genomic mutations in SARS-CoV-2 following infection in animals plays a significant role in testing the efficacy of antiviral compounds and vaccines in hamster animal models. 

## Figures and Tables

**Table 1:** **Mapping and consensus statistics of NGS data**: The SARS-CoV2 isolate of the present study used in the infection of hamsters was mapped with reference sequence NC_045512.2 and the virus isolated from the hamsters was mapped with the stock virus (used in infection) to identify the single-nucleotide polymorphisms (SNPs) and lineage. The analysis identified that the ancestral Wuhan strain (used in infection) and virus isolated from hamsters belong to lineage A.

Sample	Reference Sequence	Total Length of Consensus (bp)	No. of SNPs	Lineage Identified
Virus stock used in the infection of Hamsters	NC_045512.2	29903	8	A
Virus isolated from the infected hamster (Male)	Virus stock (used in the infection of hamsters)	29903	10	A
Virus isolated from the infected hamster (Female)		29903	10	A

**Table 2:** **Comparison of SNPs identified in the stock virus (ancestral Wuhan strain) with the reference sequence (NC_045512.2):** The SNPs identified in the viral RNA isolated from the virus stock (used in infection) were compared to the reference sequence (NC_045512.2) and the corresponding protein change is mentioned.

Position in Reference Sequence (NC_045512.2)	Reported Base in Reference Sequence (NC_045512.2)	Identified Alternate Base in Virus Stock (Used in the Infection of Hamsters)	Gene Name	Protein Change
8782	C	T	ORF1ab	Ser2839Ser
18060	C	T	ORF1ab	Leu5932Leu
21759	A	G	S	His66Arg
22296	A	G	S	His245Arg
22482	C	T	S	Thr307Ile
23606	C	T	S	Arg682Trp
23607	G	T	S	Arg682Leu
28144	T	C	ORF8	Leu84Ser

**Table 3:** **Comparison of SNPs identified in the virus isolated from hamsters (male and female) with the virus stock (ancestral Wuhan strain) used in the infection of hamsters**. Protein change remains the same in both males and females unless otherwise mentioned.

Position in Stock Virus (Used in Infection)	Reported Base in Stock Virus (Used in Infection)	Identified Alternate Base from an Infected Animal (Male)	Identified Alternate Base from Infected Animal (Female)	Gene Name	Protein Change
17827	C	A	A	ORF1ab	Gln5855Lys
21801	A	G	G	S	Asp80Gly
21849	A	-	C	S	Glu96Ala
22206	A	G	G	S	Asp215Gly
23014	A	C	C	S	Glu484Asp
23525	C	T	T	S	His655Tyr
24734	C	T	T	S	His1058Tyr
21759	G	A	A	S	Arg66His
22296	G	A	G	S	Arg245His (Male)
23606	T	C	C	S	Trp682Arg
23607	T	G	G	S	Leu682Arg

## Data Availability

The datasets generated in the current study are available from the corresponding author upon reasonable request.

## Supplementary Materials

The following supporting information can be downloaded at: https://www.mdpi.com/article/10.3390/pathogens12111328/s1, Table S1: Comparison of the SNPs identified in the virus stock used in infection and virus genome from infected female and male hamsters with the reference sequence (NC_045512.2).
