# Supplementary material for: Genomic Mutations in SARS-CoV-2 Genome following Infection in Syrian Golden Hamster and Associated Lung Pathologies
Source: Pathogens. 2023 Nov 8;12(11):1328. doi: 10.3390/pathogens12111328 (PMC10674674; doi:10.3390/pathogens12111328)
Supplement: Supplementary file 1 [file pathogens-12-01328-s001.zip › pathogens-2703893-supplementary.pdf]

Supplementary table; S1: The **c**omparison of the SNPs

Position in NC_045512.2 Reference sequence	Reported Base in NC_045512.2 sequence	Identified Alternate Base In the stock virus used in infection	Identified Alternate Base in the viral genome isolated from an infected lung (Female)	Identified Alternate Base in the viral genome isolated from an infected lung (Male)	Gene Name	Protein change
17827	C	C	A	A	ORF1ab	Gln5855Lys
21801	A	A	G	G	S	Asp80Gly
21849	A	A	C		S	Glu96Ala
22206	A	A	G	G	S	Asp215Gly
22296	A	G	-	A	S	His245Arg
23014	A	A	C	C	S	Glu484Asp
23525	C	C	T	T	S	His655Tyr
24734	C	C	T	T	S	His1058Tyr
8782	C	T	T	T	ORF1ab	Ser2839Ser
18060	C	T	T	T	ORF1ab	Leu5932Leu
21759	A	G	A	A	S	SHis66Arg
22482	C	T	T	T	S	Thr307Ile
23606	C	T	C	C	S	Arg682Trp
23607	G	T	G	G	S	Arg682Leu
28144	T	C	C	C	ORF8	Leu84Ser

Supplementary Table; S1: The **c**omparison of the SNPs identified in the virus stock (used in infection) and the virus genome isolated from the lungs of infected hamsters (female and male) with the reference sequence (NC_045512.2). The SNPs identified in the viral RNA isolated from the infected hamsters (female and male) and stock virus (used in infection) were compared with the reference sequence (NC_045512.2). The difference in genetic mutations between the reference sequence (NC_045512.2), virus stock (used in infection), and the virus genome isolated from the lungs of infected hamsters is highlighted. Protein change remains the same in both males and females.
